# Management of regional citrate anticoagulation for continuous renal replacement therapy: guideline recommendations from Chinese emergency medical doctor consensus

**DOI:** 10.1186/s40779-023-00457-9

**Published:** 2023-05-29

**Authors:** Shu-Yuan Liu, Sheng-Yong Xu, Lu Yin, Ting Yang, Kui Jin, Qiu-Bin Zhang, Feng Sun, Ding-Yu Tan, Tian-Yu Xin, Yu-Guo Chen, Xiao-Dong Zhao, Xue-Zhong Yu, Jun Xu, Emergency Medical Doctor Branch of the Chinese Medical Doctor Association, Xu-Feng Chen, Zhi Chen, Qing-Li Dou, Jian Guan, Yao-Song Gui, Zhong-Wei Huang, Xiao-Min Li, Dan-Ping Liu, Jing-Jun Lv, Yong Liu, Chuan-Yun Qian, Yi Shan, Yan Shi, Ming Sun, Hai-Ying Wu, Jian Xia, Feng Xu, Tie Xu, Xian-Liang Yan, Jian-Zhong Yang, Yong-Wu Yu, Jin-Song Zhang, Mao Zhang, Wei Zhang, Hong-Yu Zhao, Dong-Hui Zheng, Ping Zhou, Bao-Feng Zhu, Hua-Dong Zhu, Shi-Qian Shen, John Prowle, Martin Bellgardt

**Affiliations:** 1 Emergency Department, The Sixth Medical Center, Chinese PLA General Hospital, Beijing, 100048 China; 2 State Key Laboratory of Complex Severe and Rare Diseases, Emergency Department, Peking Union Medical College Hospital, Chinese Academy of Medical Science and Peking Union Medical College, Beijing, 100730 China; 3 Emergency Department, Peking University Shenzhen Hospital, Shenzhen, 518000 China; 4 Emergency Department, The First Affiliated Hospital of Kunming Medical University, Kunming, 650000 China; 5 Emergency Department, The First Affiliated Hospital of University of Science and Technology of China, Hefei, 230001 China; 6 Emergency Department, The Second Affiliated Hospital of Hainan Medical College, Haikou, 570100 China; 7 Emergency Department, The First Affiliated Hospital of Nanjing Medical University, Nanjing, 210029 China; 8 Emergency Department, Northern Jiangsu People’s Hospital, Clinical Medical College of Yangzhou University, Yangzhou, 225001 China; 9 Emergency Department and Chest Pain Center, Qilu Hospital of Shandong University, Jinan, 100005 China; 10 Shandong Provincial Clinical Research Center for Emergency and Critical Care Medicine, Jinan, 100005 China; 11 Key Laboratory of Emergency and Critical Care Medicine of Shandong Province, Qilu Hospital of Shandong University, Jinan, 100005 China; 12 Emergency Department, The Fourth Medical Center, Chinese PLA General Hospital, Beijing, 100048 China; 13 Jiangxi Provincial People’s Hospital, Nanchang, 330046, China; 14 The Second Affiliated Hospital of Shenzhen University, Shenzhen, Guangdong, 518035 China; 15 The First Hospital of Tsinghua University, Beijing, 100016, China; 16 The Affiliated Hospital and Medical School of Nantong University, Nantong, Jiangsu, 226001 China; 17 The First People’s Hospital of Lianyungang, Lianyungang, Jiangsu, 222000 China; 18 Shaanxi People’s Hospital, Xi’an, 710068 China; 19 Renmin Hospital of Wuhan University, Wuhan, 430060 China; 20 Shenzhen Hospital of Southern Medical University, Shenzhen, Guangdong, 518000 China; 21 The Second People’s Hospital of Huai’an, Huai’an, Jiangsu, 223002 China; 22 The Affiliated Suqian Hospital of Xuzhou Medical University, Xuzhou, Jiangsu, 223800 China; 23 Zhongnan Hospital of Wuhan University, Wuhan, 430071 China; 24 The Affiliated Hospital of Xuzhou Medical University, Xuzhou, 221006 China; 25 The First Affiliated Hospital of Xinjiang Medical University, Ürümqi, 830054 China; 26 Beijing Chuiyangliu Hospital, Beijing, 100022 China; 27 The Second Affiliated Hospital of Zhejiang University School of Medicine, Hangzhou, 313000 China; 28 Shengjing Hospital of China Medical University, Shenyang, 110004 China; 29 Sichuan Provincial People’s Hospital, Chengdu, 610072 China; 30 Nantong First People’s Hospital, Nantong, Jiangsu, 226001 China; 31 Anesthesia, Critical Care and Pain Medicine, Mass General Research Institute and Harvard Medical School, Boston, 02114 USA; 32 Intensive Care Medicine and Renal Medicine at the Royal London Hospital, London, E1 2AA UK; 33 Katholisches Klinikum Bochum, Bochum, 44791 Germany

**Keywords:** Continuous renal replacement therapy, Emergency, Anticoagulation, Citrate, Guideline, Expert consensus

## Abstract

Continuous renal replacement therapy (CRRT) is widely used for treating critically-ill patients in the emergency department in China. Anticoagulant therapy is needed to prevent clotting in the extracorporeal circulation during CRRT. Regional citrate anticoagulation (RCA) has been shown to potentially be safer and more effective and is now recommended as the preferred anticoagulant method for CRRT. However, there is still a lack of unified standards for RCA management in the world, and there are many problems in using this method in clinical practice. The Emergency Medical Doctor Branch of the Chinese Medical Doctor Association (CMDA) organized a panel of domestic emergency medicine experts and international experts of CRRT to discuss RCA-related issues, including the advantages and disadvantages of RCA in CRRT anticoagulation, the principle of RCA, parameter settings for RCA, monitoring of RCA (mainly metabolic acid–base disorders), and special issues during RCA. Based on the latest available research evidence as well as the paneled experts’ clinical experience, considering the generalizability, suitability, and potential resource utilization, while also balancing clinical advantages and disadvantages, a total of 16 guideline recommendations were formed from the experts’ consensus.

**Supplementary Information:**

The online version contains supplementary material available at 10.1186/s40779-023-00457-9.

## Background

Due to limited resources of critical therapy could not fully meet growing medical requirements in China, some continuous renal replacement therapy (CRRT) treatments for emergent and critically-ill patients had to be performed in emergency departments. For the success of these CRRT treatments, anticoagulant therapy was essential, which was needed to prevent clotting in the extracorporeal circulation during CRRT. Previously systemic heparin anticoagulation (SHA) had been the most widely used anticoagulation method for CRRT. However, SHA had many contraindications in emergent and critically-ill patients and also had a high complication rate limiting its application, e.g., hemorrhage or heparin induced thrombocytopenia (HIT). Therefore, some new anticoagulants and methods had been explored and developed, which included regional citrate anticoagulation (RCA). After nearly a decade of clinical practice, RCA had been shown to be safer and more effective than SHA and was now recommended as the preferred anticoagulant method for CRRT [1]. RCA was increasingly used in CRRT by emergency physicians in China. However, treatments failure and complications often occurred due to the lack of unified management guidelines in the world and inappropriate therapy in clinical practice, leading to the difficulty of wide application and popularization for RCA therapy. Therefore, it is necessary to develop standardized management guidelines for RCA therapy in China and around the world. In order to provide the latest clinical practice guidance, the Emergency Medical Doctor Branch of the Chinese Medical Doctor Association (CMDA) organized a panel of domestic emergency medicine experts and international experts to discuss RCA-related issues, and formed guideline recommendations from the experts’ consensus based on the latest available research evidence as well as the paneled experts’ clinical experience.

## Methods

This guideline was initiated and developed by the Emergency Medical Doctor Branch of the CMDA and it had been registered on the Practice Guideline Registration for Transparency (PREPARE, http://www.guidelines-registry.org) with the registration number of PREPARE-2021CN332. The guideline development group was composed of consensus expert group, in which two of them as secretary group and three consultant experts outside China, with 45 members. Experts with abundant experience in the application of RCA were selected, which were representative of regions and disciplines, covering emergency medicine, critical care medicine and guideline methodology, etc. Their main responsibilities were to screen relevant documents and gradually refine guideline recommendations. The secretary group was responsible for the organization, coordination and management of experts seminar, as well as proofreading of the guideline recommendations.

This guideline focused on the clinical management of RCA during CRRT. The English literature related to clinical management of RCA retrieval was based on a broad search of the PubMed, Medline and Cochrane databases, mainly from January 1960 to March 2023. The search terms were “continuous renal replacement therapy”, “CRRT”, “anticoagulation”, “regional citrate anticoagulation”, “RCA”, “citrate” and “Emergency Department”, combined with “AND” and “OR”. Chinese literature retrieval was performed on the CNKI, Wanfang and VIP databases.

The members of the expert group were divided into different fields, and according to the predetermined scope, the recommendations related to key clinical question, evidence and interpretation were initially drawn up. The core members of the expert group integrated the documents and wrote the full text of the guideline. The secretary group summarized the recommendations by organizing 22 discussions and revisions with the expert group members through online meetings. For each recommendation, the Delphi survey was conducted among all members of the consensus expert group by questionnaire survey. The total number of experts surveyed was 42. The design and content of the questionnaire were completed by the members of the secretary group, and were reviewed and published by the members of the expert group. The questionnaire mainly included the Likert scale score for each recommendation and the comments and suggestions area that could be filled in freely. For each recommendation, experts used the Likert scale to score, with a full score of 7 points, 7 points for very agree, 6 points for agree, 5 points for general agree, 4 points for uncertain, 3 points for not very agree, 2 points for disagree, and 1 point for completely disagree. The consensus setting: for a single recommendation, if more than 75% of experts with a score of ≥ 6 points, the experts reached a consensus for this recommendation. A total of 16 proposed recommendations had been reached consensus. Consensus strength for each recommendation was marked with “agreement degree”. Agreement degree = experts with score ≥ 6/total number of experts participating in the evaluation × 100%.

Finally, corresponding tools were used to evaluate the quality of the study and determine the documentary evidence. Based on the quality of evidence, the Grading of Recommendations Assessment, Development and Evaluation (GRADE) guidelines [2], and considering the generalizability, suitability, and potential resource utilization, while also balancing clinical advantages and disadvantages, levels of evidence and strengths of recommendation were established (Table 1).

**Table 1:** Description of evidence quality and recommendation strength

Item	Description
*Evidence quality*	
Advanced	Future studies are unlikely to have a significant impact on the current assessment and are unlikely to change current recommendations
Intermediate	Future research may have an important impact on the results of the current assessment, potentially changing the current recommendation
Low	Future research is likely to have a significant impact on the results of the current assessment, likely changing the current recommendation
*Recommendation strength*	
Strong recommendation	Most patients, doctors and policy makers adopt this approach
Medium recommendation	Most people adopt this plan, but there are still some people who do not adopt it. It is necessary to make a decision reflecting the values and wishes of patients based on their specific situation
Weak recommendation	The evidence is insufficient and a decision needs to be made by patients, physicians and policy makers

### Recommendation statements for management of RCA during CRRT

#### Advantages of RCA in CRRT anticoagulation therapy

##### Recommendation 1

*RCA is recommended as the preferred anticoagulation method for emergent and critically-ill patients receiving CRRT if without contraindication. (A*greement degree: 92.9%, *Evidence quality I, Recommendation strength A).*

**Remarks**

SHA was once the standard anticoagulation for CRRT, which helped ensure that blood in extracorporeal circulation did not coagulate. Unfortunately, SHA has many disadvantages, such as contraindications in patients with active bleeding or those with a high risk of bleeding, weight-based (individualized) dosing, many interactions affecting anticoagulant monitoring, a high incidence of bleeding complications, and HIT.

RCA has a longer extracorporeal circulation circuit lifespan than SHA. The duration of the circuit (especially the filter) is associated with many variables, such as a patient’s coagulation status, the location of their vascular access, the CRRT mode, and parameter settings. Studies comparing the effects of different anticoagulation methods on the lifespan of the circuit have significant heterogeneity. A Meta-analysis of 30 studies comparing the effects of RCA and SHA on CRRT circuit lifespan showed that filter coagulation was significantly delayed in the RCA group compared with the SHA group (mean difference 15.69 h, 95%CI 9.30–22.08). In addition, a subgroup analysis of different modes also showed that the RCA group had a longer circuit lifespan [3]. In another prospective multicenter randomized controlled study, RCA significantly reduced system downtime in the first 72 h of treatment (1 h vs. 3 h), had fewer treatment interruptions due to coagulation (24% vs. 51%), or filter replacements (9% vs. 30%) when compared with SHA [4]. A longer filter lifespan and fewer treatment interruptions mean more efficient CRRT delivery [5].

Compared with SHA, RCA has a lower incidence of bleeding and less need for blood transfusions [6]. Two meta-analyses compared the incidence of bleeding during CRRT between SHA and RCA, and showed a lower risk of bleeding in the RCA group (*RR* = 0.31, 95%CI 0.19–0.51; *RR* = 0.34, 95%CI 0.17–0.65, respectively) [3, 7]. In a study of critically-ill patients with acute kidney injury after cardiac surgery, RCA not only prolonged the filter lifespan, but also significantly reduced the need for transfusions compared with SHA (0.29 vs. 0.62 blood units/d, *P* = 0.017) [8]. Since blood in the CRRT extracorporeal circulation circuit (about 150 to 200 ml of blood) will often fail to return if there is clotting in the extracorporeal circuit or filter, it makes sense that fewer blood transfusions are needed with RCA compared with SHA [9].

Other advantages of RCA include [4, 10–12]: 1) No side effects such as leukopenia and thrombocytopenia associated with using heparin; 2) Citrate is a physiological substance in the body with biocompatibility advantages; 3) Some studies have shown that low serum calcium during RCA use may be beneficial to inflammatory responses; 4) Due to the lower filter replacement ratio, there may be a better total cost/benefit for using RCA.

Nevertheless, strong evidence about the impact of RCA vs. SHA on survival is still lacking. Two multicenter randomized studies enrolled 170 and 212 CRRT patients, respectively, to compare the impact on survival between using SHA or RCA and found no statistical difference in survival between the two groups [13, 14]. Furthermore, two meta-analyses compared mortality between the RCA and SHA groups, and also found no advantage for RCA [3, 15]. The ongoing RICH study is the largest prospective multicenter randomized clinical trial to date and aims to add further evidence to address the relationship between CRRT anticoagulant regiments and patient outcomes [16].

Citrate is contraindicated in patients with liver disease or shock states in Kidney Disease Improving Global Outcomes (KDIGO) guidelines [1]. Recent studies suggested that liver disease and shock states were not absolute contraindications for RCA, and these would be discussed in detail below. Regardless, RCA use was at high risk for patients with liver disease and shock, it should be treated with caution.

#### Principles of RCA

##### Recommendation 2

*It is recommended to set initial citrate dose based on blood flow rate, and the citrate dose should be appropriately adjusted according to the monitoring value of filter ionized calcium (iCa). (A*greement degree: 97.6%,* Evidence quality I, Recommendation strength A).*

**Remarks**

Citrate is widely found in various tissues and fluids in the human body. It is not only an important component of the skeletal system but also an intermediate product of the metabolism of glucose, lipids, and some amino acids. It plays an important role in energy metabolism. In normal circumstances, the serum concentration of citrate is extremely low, about 0.1 mmol/L, mainly in a form of stable soluble calcium citrate.

**Fig. 1:**
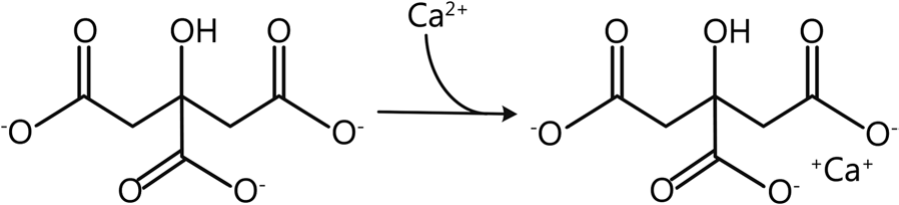
Chelation reaction of the trivalent anion of Citrate^3−^ with an ionized calcium

Citrate contains three carboxyl groups that can dissociate into the trivalent anion of Citrate^3−^. Because of the characteristics of its structure, two negatively charged carboxyl groups in Citrate^3−^ can chelate with divalent cations such as iCa or magnesium in blood and form monovalent anions like calcium citrate or magnesium citrate (Fig. 1). The chelation reaction is rapid and the chelate does not dissociate spontaneously in the circulation. The space distance between the two positive charges of iCa matches better with the distance between the two negative charges of the Citrate^3−^ carboxyl groups, resulting in stronger citrate-calcium complexes (CCC).

When citrate is infused into the blood, it rapidly chelates the iCa in the blood to form CCC (equivalent to changing the iCa to binding calcium), thus reducing the level of iCa in the blood. iCa also called coagulation factor IV which is vital for multiple steps of coagulation process and platelet interactions. In the endogenous coagulation pathway, iCa can assist in activating factor XI, and activate factor X together with factor VIII and activated factor IX. In the exogenous coagulation pathway, factor X is activated by iCa, factors III and VII. In the common pathway, fibrinogen can be converted into fibrin monomers by assistance of iCa, factor V and activated factor X. In addition, it can also assist in activating factor XIII and continue to assist factor XIII in transforming soluble fibrin monomers into stable fibrin polymers. The exogenous and endogenous coagulation pathway steps would be blocked if the blood level of iCa decreases obviously. Under physiological conditions, the concentration of serum iCa is 1.0–1.2 mmol/L. The blood coagulation will not occur if the blood level of iCa is reduced to below 0.35–0.4 mmol/L [17, 18].

**Fig. 2:**
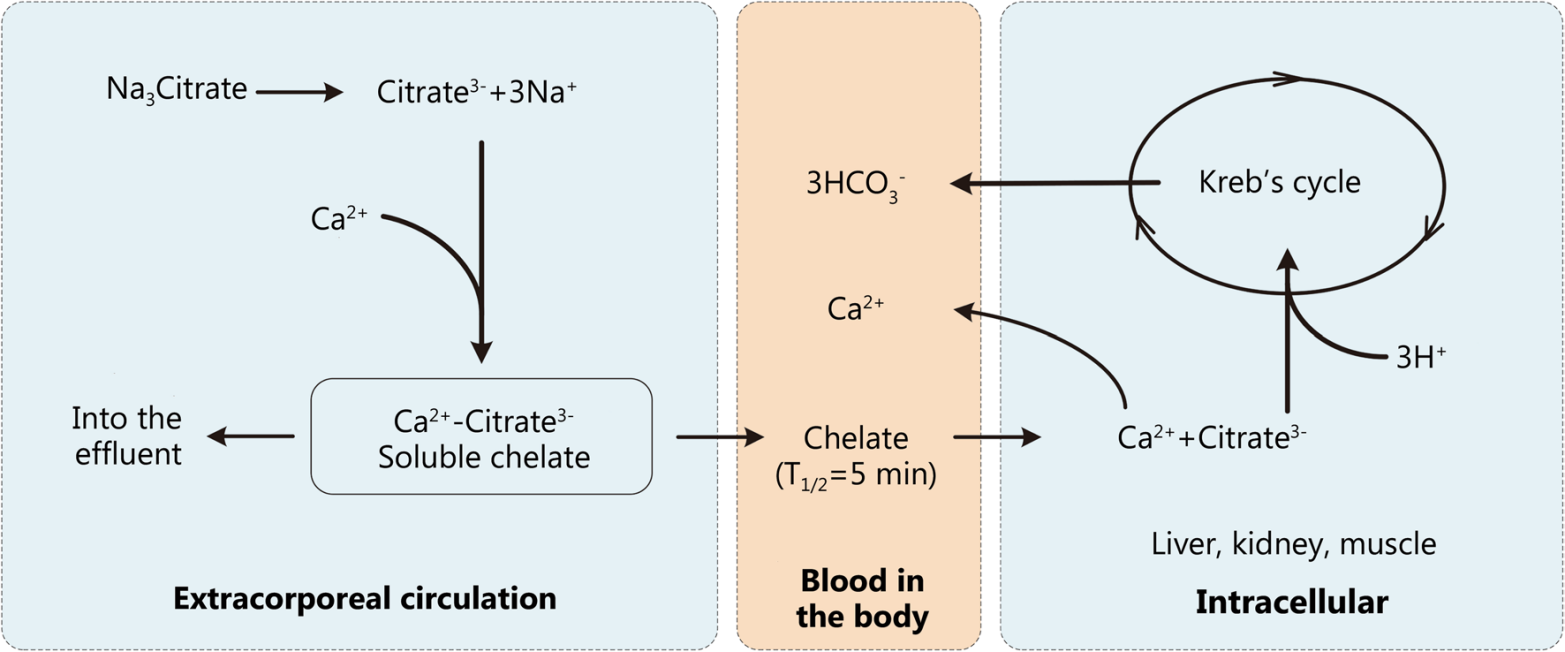
Schematic diagram of citrate metabolism. When trisodium citrate is infused into the extracorporeal circulation, citrate-calcium complexes (CCC) is formed by the chelation reaction of ionized calcium (iCa) and Citrate^3−^. iCa is rapidly reduced to prevent clotting. About 30–60% of CCC molecules enter the effluent through the permeable membrane depending on different continuous renal replacement therapy (CRRT) modes and therapeutic doses. The residual CCC molecules return to the systemic circulation and are metabolized rapidly in body cells. The half-life of CCC under physiological conditions is only 5 min. CCC is eventually decomposed into bicarbonate through the tricarboxylic acid oxidation cycle (one molecule of citrate yields three molecules bicarbonate), and the iCa is released back into the blood

**Fig. 3:**
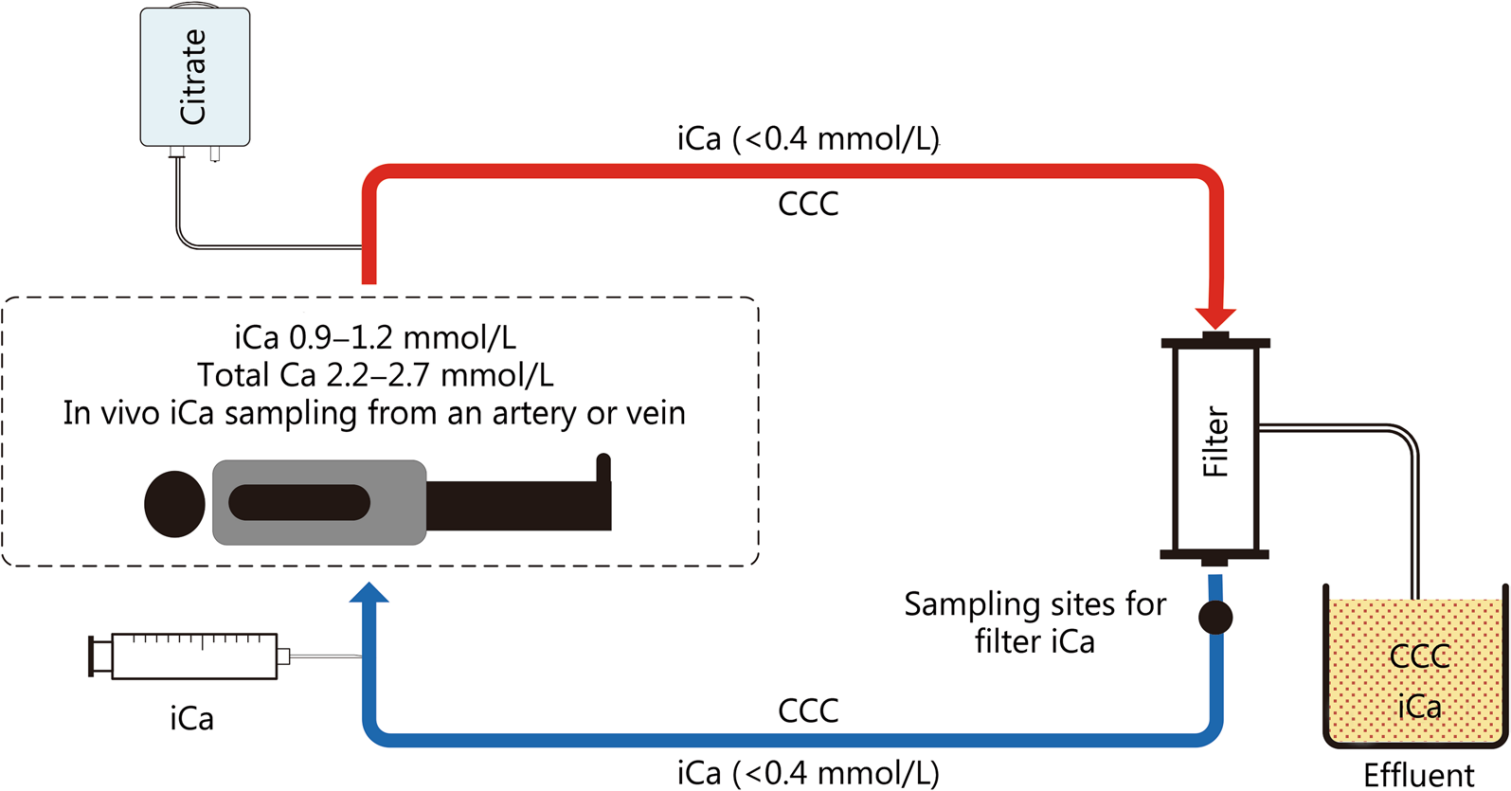
Schematic diagram of RCA in the CRRT system. Citrate is infused into the extracorporeal circulation before filter. Citrate-calcium complexes (CCC) is formed by the chelation reaction of ionized calcium (iCa) and Citrate^3−^. Partial CCC and iCa are removed by the filter. As a result, iCa in the filter is rapidly reduced to below 0.4 mmol/L, thus preventing clotting. The concentration of iCa in the filter is routinely monitored at the site behind the filter. Before the blood is returned to the body, additional iCa is infused to the blood in order to replenish the calcium removed to effluent by the filter. RCA regional citrate anticoagulation, CRRT continuous renal replacement therapy

During CRRT, when citrate is infused at the access end of the extracorporeal circulation, the concentration of calcium in the extracorporeal circulation is rapidly reduced to below 0.4 mmol/L, thus preventing clotting. The negative univalent CCC formed by the chelation reaction is a small molecule (298 Dalton) with good water solubility and a sieving coefficient of about 1, which can be rapidly removed by the filter through its semi-permeable membrane [19, 20]. Due to different CRRT modes and therapeutic doses, about 30–60% of CCC molecules will enter the effluent through the permeable membrane. The CCC molecules which are not removed via the hemofilter enter the systemic circulation and are first metabolized into citric acid rapidly in body cells. Citric acid is metabolized through the tricarboxylic acid cycle, which is oxygen dependent and mainly occurs in organs with high mitochondrial content. In physiological conditions, it is mainly metabolized in liver and a small portion is metabolized in skeletal muscles. The half-life of CCC under physiological conditions is only 5 min. CCC is eventually decomposed into bicarbonate through the tricarboxylic acid oxidation cycle (one molecule of citrate yields three molecules bicarbonate), and the iCa is released back into the blood. Meanwhile, the unchelated calcium ions are also partially removed from the circuit blood into the effluent (Fig. 2). As a result, severe hypocalcemia can occur if ongoing supplementation of iCa is not provided. Therefore, adequate amounts of iCa must be supplied directly to the patient intravenously or through the return end of extracorporeal circulation circuit to maintain physiological iCa level. RCA anticoagulation is thus achieved only in the CRRT circuit (i.e., regional anticoagulation) with little interference with in vivo coagulation processes (Fig. 3).

When RCA is used, citrate is added at the access end  of extracorporeal circuit to ensure a citrate concentration of 3–4 mmol/L (i.e., 3–4 mmol citrate per 1 L of whole blood) [9, 21–25]. The flow rate of citrate is then adjusted by monitoring the concentration of iCa in the filter (hereinafter referred to as iCa in the filter, with the sampling site residing behind the filter, as shown in Fig. 3), so as to ensure the effectiveness of anticoagulation with the concentration of iCa in the filter ranging from 0.2 to 0.4 mmol/L. In order to supplement the calcium lost in the effluent through the filter membrane, extra iCa is infused in the return end of the extracorporeal circuit or directly to patients via central vein to ensure normal serum calcium in the patient. It is very important to monitor the concentration of iCa in patients’ systemic circulation (hereinafter referred to as iCa in the body, sampled from the patient’s artery or vein).

#### Parameter settings for RCA

##### Setting the initial flow rate for citrate and scheme adjustment

###### Recommendation 3

*It is preferable to use a calcium-free solution as a replacement fluid in predilution continuous veno-venous hemofiltration (CVVH) or as a dialysate in continuous veno-venous hemodialysis (CVVHD). If a replacement fluid containing calcium is used and predilution CVVH or CVVHD mode is selected during RCA, it is recommended to adjust the citrate dose appropriately to achieve targeted filter iCa concentration. (A*greement degree: 97.6%,* Evidence quality III, Recommendation strength B).*

###### Recommendation 4

*The recommended target value of iCa in the filter is 0.2*–*0.4 mmol/L, but the target value should be individually adjusted according to the treatment situation. (A*greement degree: 100.0%, *Evidence quality II, Recommendation strength C).*

**Remarks**

Currently, there are two forms of Citrate^3−^ solutions available for anticoagulation: one is only used as an anticoagulant (known as citrate anticoagulant). It includes 4% trisodium citrate (TSC) solution and acid-citrate-dextrose formula A (ACD-A). ACD-A is a type of fluid used for blood preservation but is now less commonly used in CRRT for anticoagulation. The other form of solution is a kind of buffer solution containing Citrate^3−^ (balanced solution) which can be used for anticoagulation and as a predilution replacement fluid, known as citrate replacement solution. Currently, there are mainly manual citrate-based replacement solution (balanced solution) and commercial solutions (e.g., prismocitrate 10/2, prismocitrate 18/0). Citrate-based replacement solution cannot be used for postdilution or dialysis. Citrate^3−^ contents, electrolyte concentration and physicochemical properties of the above solutions are listed in Table 2 [26].

**Table 2:** Composition and properties of solution containing Citrate^3−^

Composition and characteristics	Citrate anticoagulant		Citrate replacement solution		
	4% TSC	ACD-A	TSC + citric acid + NaCl	TSC + NaCl	Citrate balanced solution
Na^+^ (mmol/L)	420	224	136	140	140–159
Citrate^3−^ (mmol/L)	136	113	10 (+ 2)^a^	18	13–20
Glucose (mmol/L)	None	139	None	None	± 5
Cl^−^ (mmol/L)	None	None	106	86	99–108
Mg^2+^ (mmol/L)	None	None	None	None	0–0.75
SIDe^b^ (mmol/L)	420	224	30	54	33–54
SIDe of every mmol Citrate^3−^	3.00	2.00	3.00	3.00	2.25–3.00

^a^Two Citrate^3−^ are from citrate; ^b^SIDe: Strong ion difference calculated after metabolism of citrate, by [(Na^+^  + K^+^  + Ca^2+^  + Mg^2+^)—Cl^−^]; *TSC* trisodium citrate, *ACD-A* citrate-glucose anticoagulant A, *NaCl* normal saline, *SIDe* effective strong ion difference

There is a corresponding relationship between citrate flow rate and blood flow rate. The initial flow rate of citrate should be determined based on the blood flow rate to reach a citrate concentration of about 3–4 mmol/L in the extracorporeal circulation, as shown in Table 3. Considering the adequacy of initial anticoagulation and the practical experience of each expert, the guideline suggests that if calcium free replacement fluid is used, the initial flow rate of citrate solution should be: 1) ACD-A (ml/h) = blood flow rate (ml/min) × 1.8; 2) 4% TSC solution (ml/h) = blood flow rate (ml/min) × 1.5. If replacement fluid containing calcium is used and predilution CVVH or CVVHD mode is chosen (iCa concentration in commercial replacement fluid is usually 1.6 mmol/L in China, see Additional file 1: Table S1), the flow rate of citrate solution should be increased to chelate iCa in the replacement fluid. The initial flow rate is empirically suggested to be as follows: 1) ACD-A (ml/h) = blood flow rate (ml/min) × 2.0; 2) 4% TSC solution (ml/h) = blood flow rate (ml/min) × 1.7.

**Table 3:** Relationship between the flow rates of citrate and blood

When the blood flow rate is 150 ml/min, hourly conversion is 9000 ml/h (= 9.0 L/h)		
Target blood concentration of citrate	3 mmol/L	4 mmol/L
Required amount of citrate	3 × 9.0 = 27.0 mmol/h	4 × 9.0 = 36.0 mmol/h
3% ACD-A solution (113 mmol/L)	27.0/113 = 0.239 L/h ≈ 240 ml/h	36.0/113 = 0.319 L/h ≈ 320 ml/h
4% TSC solution (136 mmol/L)	27.0/136 = 0.198 L/h ≈ 200 ml/h	36.0/136 = 0.264 L/h ≈ 260 ml/h

To achieve the ideal anticoagulant strength, the ratio of 3% ACD-A solution to blood flow is about 1.60–2.13 (ml/h vs. ml/min); the ratio of 4% TSC solution to blood flow is about 1.33–1.73 (ml/h vs. ml/min). *ACD-A* citrate-glucose anticoagulant A, *TSC* trisodium citrate

Filter calcium levels should be closely monitored during the first 24 h of treatment (usually measured with a blood gas analyzer). It is recommended to conduct the first detection 30 min after the start of treatment, and then monitor iCa every 2 h for 4 consecutive times. Adjust the flow of citrate according to the level of iCa in the filter, and then monitor it once every 4 h for 4 consecutive times after it is deemed stable. If the treatment goes well, monitoring can be changed to once every 6 h after 24 h, i.e., (30 min) → (q2 h × 4) → (q4 h × 4) → (q 6 h × 4). According to the measured concentration of iCa in the filter, the citrate flow rate can be adjusted by referring to Table 4.

**Table 4:** Flow of citrate infusion and adjustment scheme

iCa in the filter (mmol/L)	Flow of 4% TSC infusion	Flow of ACD-A infusion
≤ 0.20	Reduce 10 ml/h	Reduce 12 ml/h
0.20–0.40	Unchanged	Unchanged
0.40–0.50	Increase 10 ml/h	Increase 12 ml/h
> 0.50	Increase 20 ml/h	Increase 24 ml/h

*iCa* ionized calcium, *ACD-A* citrate-glucose anticoagulant A, *TSC* trisodium citrate

It should be emphasized that adequate anticoagulation in the early CRRT phase is very important because the cascade of amplification reactions may accelerate clotting once microthrombi are formed in the filter. Therefore, iCa should be closely monitored after the start of treatment, and the program should be actively adjusted to achieve stabilization of anticoagulation as soon as possible.

From the perspective of the principles of using RCA, factors determining the dose of citrate include: 1) Blood flow rate. The citrate flow rate must match the blood flow rate to ensure a stable anticoagulant concentration in the extracorporeal circulation and should be adjusted accordingly after a change in the blood flow rate. 2) The site where replacement fluid is added. If a calcium-free replacement fluid is used in predilution mode, the blood entering the filter will be diluted, so that the iCa concentration in the filter will decrease, and the demand for citrate will decrease accordingly. 3) Composition of replacement fluid. Whether the replacement fluid contains calcium and magnesium also affects the flow rate of citrate. If calcium-containing replacement fluid is used in predilution mode, the amount of iCa entering the filter will increase, requiring more citrate to chelate them. If calcium-containing replacement fluid is used in postdilution mode, the impact on the amount of citrate will be small, but may increase the risk of coagulation in the post-filter air trap chamber. The blood sampling location of the iCa in the filter is usually before the infusion site of the postdilution replacement fluid, and the post-filter air trap chamber is usually located behind the input point of the postdilution replacement fluid, therefore the actual iCa concentration in the post-filter air trap chamber is higher than the measured value. 4) Individual anticoagulation intensity target. The anticoagulant intensity required in vitro may vary depending on the coagulation state of the patient. There is a huge individual difference in the iCa threshold to prevent blood coagulation, such as satisfactory anticoagulation is achieved in some patients when iCa in the filter are only less than 0.5 mmol/L, while in some others, they are required to be 0.2 mmol/L strictly [27–31]. In addition, error measurement of iCa in post-filter also prompts that anticoagulation intensity should not be set only based on the level of iCa. There are ongoing concerns of technologies when measuring "post-filter" iCa. These technologies have been developed to measure ionized Ca in the physiologic range, however there is great uncertainty in the accuracy of measurement of iCa when measuring in subphysiologic range using blood gas analyzers. Therefore although the target range of 0.2–0.4 mmol/L of iCa in the filter is appropriate for most patients, in practice the target value should be individualized according to the actual treatment process rather than being restricted to this range [32]. Especially when clotting start to occur in the filter, it would call for empirical increase of citrate infusion rate to minimize clotting propensity despite the level of iCa is in the range in post-filter.

##### Calcium supplementation and scheme adjustments

###### Recommendation 5

*Calcium supplementation is recommended to maintain *in vivo* physiological levels during RCA (A*greement degree: 100.0%, *Evidence quality II, Recommendation strength A). Calcium supplementation can be administered through the return end of the extracorporeal circulation circuit or directly through the central vein, rather than directly through a peripheral vein (A*greement degree: 100.0%, *Evidence quality III, Recommendation strength B).*

###### Recommendation 6

*If calcium-free replacement fluid is used during RCA, it is recommended that the initial flow of 5% calcium chloride infusion be set to an effluent flow rate divided by 200 or 10% calcium gluconate infusion be set to an effluent flow rate divided by 125; If a calcium-containing replacement fluid is used and predilution CVVH or CVVHD mode is selected, an initial flow of 5 ml/h for 5% calcium chloride infusion or 8 ml/h for 10% calcium gluconate infusion is recommended. (A*greement degree: 100.0%, *Evidence quality III, Recommendation strength C).*

###### Recommendation 7

*During RCA, it is recommended to adjust the calcium supplementation rate according to the monitoring *in vivo* value of iCa (A*greement degree: 100.0%, *Evidence quality II, Recommendation strength A). The recommended target *in vivo* value of iCa is 0.9*–*1.1 mmol/L (A*greement degree: 90.4%, *Evidence quality II, Recommendation strength C).*

**Remarks**

In order to ensure patient safety, it is necessary to closely monitor the in vivo iCa level during RCA to prevent serious abnormal blood calcium levels. In vivo iCa monitoring should be obtained from the patient’s body (either arterial or venous sampling) or from the access end of the extracorporeal circulation circuit (prior to the site of citrate addition). Some machines provide a special blood sampling site at the access end of the extracorporeal circulation circuit.

The physiological level of in vivo iCa is 1.0–1.2 mmol/L. With the exception of some situations (e.g., hyperkalemia), hypocalcium-related symptoms usually do not occur with iCa concentration above 0.8 mmol/L. If the optimal target value of iCa is set slightly below a typical physiological level, such as 0.9–1.0 mmol/L, the amount of citrate required may be reduced. In addition, a slightly lower calcium target may help to reduce systemic inflammation [18, 33–36].

In China, 5% calcium chloride and 10% calcium gluconate are commonly used for iCa supplementation, and the difference in iCa concentration between them is about 1.5 times (iCa concentration in 5% calcium chloride solution is about 1.5 times that of 10% calcium gluconate, see Table 5). Additional iCa supplements can be administered at the return end of the extracorporeal circulation circuit or directly through a central venous catheter. Because calcium chloride is a strong vascular irritant and extravascular leakage that may occur during infusions can cause tissue necrosis, peripheral venous infusion should be avoided. 10% calcium gluconate infusion also carries a similar risk and is not recommended for peripheral infusion.

**Table 5:** Commonly used intravenous iCa supplements and their concentrations

Calcium supplements	Mass concentration (g/L)	Molecular weight (Da)	iCa concentration (mmol/L)
5% calcium chloride (dihydrate)	50	147	340
10% calcium gluconate (monohydrate)	100	448	224

*iCa* ionized calcium

As for setting the initial flow rate of calcium supplementation, there are some empirical formulas to estimate the starting flow rate of calcium supplementation according to the citrate flow rate. However, considering the metabolic process of citrate, the rate of calcium supplementation is not necessarily related to the flow rate of citrate, but is determined by the total amount of calcium lost through the effluent. Due to the complex balance between binding calcium and iCa in the circulation when partial iCa is chelated by citrate during CRRT, and the difference in sieving coefficient between calcium citrate and iCa, it is difficult to accurately calculate the dose of calcium supplement through therapeutic parameters. Studies to help determine the best initial rate of calcium supplementation are also lacking. This guideline recommends using empirical formulas for the initial settings. If a calcium-free replacement fluid is used, it is recommended that the initial flow rate of 5% calcium chloride infusion is set to an effluent flow rate divided by 200, or, if using a 10% calcium gluconate infusion, it should be set to an effluent flow rate divided by 125. If a calcium-containing replacement fluid is used and a predilution CVVH or CVVHD mode is selected, an initial flow rate of 5 ml/h if using a 5% calcium chloride infusion or 8 ml/h if using a 10% calcium gluconate infusion is recommended. This empirical initial value may not be appropriate for all patients, but if standardized iCa monitoring is performed, it should not result in severe blood calcium abnormalities during the initial few hours because of the considerable buffering capacity of the calcium pools in the body. It should be emphasized that the above empirical values are only for making initial settings, and the calcium supplement rate must be further adjusted according to the level of in vivo iCa. It is suggested to monitor the in vivo iCa level according to the previously stated plan of (30 min) → (q2 h × 4) → (q4 h × 4) → (q6 h × 4) after starting treatment, and adjust the calcium supplement rate according to the plan in Table 6.

**Table 6:** Adjustment scheme for the iCa supplemental infusion

In vivo iCa (mmol/L)	5% calcium chloride	10% calcium gluconate
≥ 1.2	Reduce 2 ml/h	Reduce 3 ml/h
≥ 1.0	Reduce 1 ml/h	Reduce 1.5 ml/h
≥ 0.9	Unchanged	Unchanged
≥ 0.8	Increase 1 ml/h	Increase 1.5 ml/h
< 0.8	After 0.1 ml/kg IV, increase 2 ml/h	After 0.15 ml/kg IV, increase 3 ml/h

iCa should be measured in vivo (sampling from artery or vein) or at the access end of the extracorporeal circulation circuit. *IV* intravenous injection, *iCa* ionized calcium

CCC formed by chelating iCa is a soluble small molecule substance, part of which enters the effluent and means there will be a net loss of chelated calcium, while the remainder (i.e., unchelated iCa) is returned to the body. Some of the unchelated iCa in the extracorporeal circulation also pass through the filter into the effluent. Therefore, the amount of iCa that needs to be supplemented is actually the total net loss of calcium in the effluent. All the factors that affect the permeability of the semi-permeable membrane and the flow rate of effluent can affect the amount of iCa supplementation required. Accordingly, the factors influencing iCa supplementation include: 1) All factors that increase the flow rate of the replacement fluids (pre-dilution + post-dilution + dialysate) will increase calcium loss, and changes to these parameters may affect the calcium supplementation rate. 2) When using a replacement fluid containing calcium, although the ionized calcium concentration in commercial replacement fluid (e.g., 1.6 mmol/L in China, See Additional file 1: Table S1 for more details) is often higher than physiological concentration in blood, calcium supplementation may still be required, but at a reduced amount compared to the use of calcium-free replacement fluids [24, 25, 37–39]. 3) As the treatment time lengthens, the efficiency of the semi-permeable membrane gradually decreases, and the loss of calcium through the filter will be reduced if other parameters remain unchanged, so the amount of supplemental calcium also decreases slightly. In addition, it should be noted that if the rate of iCa supplementation needs to be continuously increased during treatment to maintain a normal serum iCa level, the possibility that serious complications should be considered. (see below).

#### Monitoring of RCA

Although RCA has many advantages over SHA, it is not a perfect anticoagulation method, and serious complications may occur if monitoring is not standardized. The main complications associated with RCA include acid–base balance and electrolyte disturbances. Lacking accurate understanding of the mechanisms of complications can lead to inappropriate management. The metabolic complications associated with RCA are summarized in Table 7.

**Table 7:** Pathogenesis and management of RCA-related metabolic complications

Item	Citrate accumulation	Alkali overload (including citrate overload)	Insufficient base delivery
Mechanism	CCC metabolism is inhibited and CCC persists in the systemic circulation	CCC metabolism is normal, alkali load exceeds demand	CCC metabolism is normal, alkali load cannot meet demand
Acidosis or alkalosis	Metabolic acidosis	Metabolic alkalosis	Metabolic acidosis
Total-Ca/iCa	Increased (≥ 2.5)	Normal (< 2.5)	Normal (< 2.5)
iCa	Decreased	No change	No change
Calcium supplement	Increased	No change	No change
Consequences	Potentially lethal	Benign, easy to correct	Benign, easy to correct
Incidence rate	Rare	Common	Infrequent
Management	Manage the cause, or reduce citrate infusion rate, or consider other anticoagulant strategies	Reduce additional alkali, or increase the flow rate of replacement fluid or dialysate, or reduce citrate infusion rate	Add additional alkali, or decrease the flow rate of replacement fluid or dialysate, or increase citrate infusion rate

*RCA* regional citrate anticoagulation, *iCa* ionized calcium, *CCC* citrate-calcium complexes

##### Metabolic alkalosis

###### Recommendation 8

*Metabolic alkalosis is the most common complication of RCA, and the primary management strategy is to decrease extra bicarbonate infusion, or increase the flow rate of replacement fluid or dialysate if without extra bicarbonate infusion. (A*greement degree: 92.9%, *Evidence quality II, recommendation strength C).*

**Remarks**

Metabolic alkalosis is a relatively common and an easily controlled complication of using RCA, with an overall incidence of 29–50% [40, 41]. There are two sources of bases related to RCA, one is derived from exogenous bicarbonate supplementation, and the other is metabolite of CCC entered in the systemic circulation. When CCC is completely metabolized, it will release iCa and produce HCO_3_^−^ at the same time. Taking 4% TSC solution as an example, infusing 200 ml of 4% TSC solution is equivalent to infusing 137 ml of 5% NaHCO_3_ solution. When the total provision of bicarbonate exceeds the therapy requirement, metabolic alkalosis will occur. If without exogenous bicarbonate infusion, metabolic alkalosis still exists, this situation is called “citrate overload”, which imply excessive CCC entering the systemic circulation and it can be completely metabolized and then produce excessive HCO_3_^−^, leading to metabolic alkalosis. “Citrate overload” is a sign of excessive citrate administration, or more frequently, of low clearance in the hemofilter [42].

The risk of metabolic alkalosis varies with different anticoagulants containing citrate. According to Stewart’s principle of acid–base equilibrium [43–46], alkalization occurs when the strong ion difference (SID) in plasma increases after citrate is metabolized in the body. Each 1 mmol of citrate provided by 4% TSC metabolizes into 3 mmol of SID, while only 2 mmol of SID are produced by ACD-A. Therefore, under the same anticoagulation strength (same amount of citrate provided), 4% TSC has a stronger alkalizing effect on plasma, leading to more cases of metabolic alkalosis. In addition, the use of different replacement fluids or dialysate may also have an impact on metabolic alkalosis. When lactate replacement solution fluid (with a lactate concentration usually of 25–45 mmol/L, and one molecule of lactate in vivo produces one molecule of HCO^3−^) and RCA (also producing bicarbonate) are used together, metabolic alkalosis is more likely to occur because excessive alkali produced by metabolization of lactate and citrate in the body is over the therapeutical requirements during CRRT.

The common treatment strategies for metabolic alkalosis include: 1) If other alkali agents (e.g., 5% NaHCO_3_) are being used, consider reducing or discontinuing such infusions; 2) Increase the flow rate of replacement fluid or dialysate to increase the proportion of CCC cleared by the semi-permeable membrane, and also increase the removal of circulating bicarbonate; 3) If without extra bicarbonate infusion and the dose of replacement fluid or dialysate is enough, it can be considered to reduce the infusion of citrate under the premise of ensuring anticoagulation effect. Reduce the flow rate of blood to match the reduced flow rate of citrate if necessary. It should be noted that lowering blood flow rate can lead to an increase in the filtration fraction of postdilution mode of CVVH/CVVHDF, which may increase the risk of thrombosis in the filter; 4) If the lactate replacement fluid is used, consider changing the replacement fluid formulation; 5) The filter should also be replaced when CCC clearance drops if caused by a decrease in filter efficiency. Infusion of saline is not recommended for the treatment of RCA-related metabolic alkalosis unless the patient also has evidence of having insufficient volume.

##### Metabolic acidosis

###### Recommendation 9

*The accumulation of citrate should be monitored during RCA. The following indicators are suggested as early warning signs of citrate accumulation: 1) Total calcium/iCa is* > *2.25 and increasing; 2) Gradually worsening ionized hypocalcemia; 3) The amount of iCa supplementation is gradually increasing. (A*greement degree: 95.2%, *Evidence quality II, recommendation strength B).*

###### Recommendation 10

*The following actions are suggested for managing citrate accumulation: 1) Optimize hemodynamics and tissue perfusion to correct hypoxia and shock; 2) Reduce the rate of citrate infusion; 3) Finally, change anticoagulant methods if the above treatments are ineffective. (A*greement degree: 95.2%, *Evidence quality III, Recommendation strength C).*

###### Recommendation 11

*When metabolic acidosis related to RCA during CRRT occurs, the following treatment strategies are suggested: 1) Check for any evidence of citrate accumulation. If citrate accumulation is the cause, follow Recommendation 10; 2) Add additional base, such as NaHCO*_*3*_*; 3) Reduce the replacement fluid or dialysate flow rate; 4) Increase infusion rate of citrate. (A*greement degree: 90.5%, *Evidence quality III, Recommendation strength B).*

**Remarks**

Metabolic acidosis may occur during RCA, involving two different mechanisms with different management strategies.

***Citrate accumulation***

The body’s ability to metabolize citrate is limited, but it’s still not clear what the maximum is. With an increase in the Citrate^3−^ infusion rate during RCA, CCC concentration in the systemic circulation also increases. Limited data shows that a systemic circulation CCC concentration of less than 2–3 mmol/L may be safe [47–51]. Under general anticoagulant doses, the infusion rate of Citrate^3−^ is less than 40 mmol/h, and nearly 50% of the CCC is lost in the effluent through the filter. The concentration of CCC that finally enters the body’s circulation is generally less than 2 mmol/L, which has a large margin of safety [48–51]. However, if a patient’s tricarboxylic acid cycle is significantly inhibited, such as in the presence of severe hypoxia or hypoperfusion, citrate accumulation (that is, large amounts of CCC in the bloodstream) can occur as a result of significant metabolic impairment.

Citrate accumulation is a rare but potentially fatal complication during RCA. In fact, CCC itself is not toxic, but accumulation of CCC usually occurs in patients with significant impairment of the tricarboxylic acid cycle, often accompanied with severe disease states such as hypoxia, organ failure, hypoperfusion, and lactic acidosis. In a retrospective study including 1070 patients with RCA-CRRT, citrate accumulation occurred in 32 patients, with an overall incidence of 2.99% (32/1070), and none of these 32 patients survived [52]. Another prospective study including 100 RCA-CRRT patients at high risk of bleeding after surgery showed that the incidence of citrate accumulation was 1% [41].

At present, plasma citrate levels cannot be routinely measured in most clinical laboratories, therefore citrate accumulation can only be predicted by indirect signs. The first sign is “citrate gap” which is the lingo in contemporary practice of CRRT referring to the difference between total and iCa in the United States. The total calcium level is the total of all calcium, including the ionized, protein bound and citrated calcium forms. With progressive systemic citrate accumulation, the increased citrate combines with more ionized calcium, which lead to an increase of citrated calcium, and meanwhile a decrease occurs in CCC metabolism in the systemic circulation. When the iCa released by CCC decreases, it may result in lower level of iCa. Therefore, increased total calcium, increased “citrate gap”, progressive ionized hypocalcemia or a gradually increasing requirements for calcium supplementation are the signs of citrate accumulation. Moreover, similar to the concept of “citrate gap”, a gradually increasing total-Ca/iCa ratio is highly suggestive of progressive citrate accumulation, and a ratio ≥ 2.5 often indicates the presence of significant citrate accumulation. Studies showed that total-Ca/iCa ratio is highly correlated with plasma CCC level and might be the most reliable indicator [42, 53–56]. Furthermore, as CCC metabolism decreases, plasma bicarbonate also decreases. New or worsening metabolic acidosis may be another sign of citrate accumulation. Since almost all such patients had severe organ failure and persistent hyperlactatemia, they presented as metabolic acidosis with a high anion gap. However, neither hyperlactatemia nor high anionic gap metabolic acidosis had a consistent association with plasma CCC level, and neither could reliably predict or detect citrate accumulation [53, 54]. They can only indicate a high risk for citrate accumulation.

In summary, the recommendation suggests the following indicators as warning signs of citrate accumulation [55, 56]: 1) Total-Ca/iCa ratio > 2.25 with dynamic increases; 2) Gradually worsening ionized hypocalcemia; 3) Gradually increasing requirements for intravenous calcium supplementation. It is recommended that total-Ca level be measured at least once a day to calculate the total-Ca/iCa ratio. The occurrence of citrate accumulation does not mean that anticoagulant methods must be changed. A considerable number of patients can be successfully treated by reducing the CCC load [57]. The suggested treatments are as follows: 1) Down-regulating blood flow and thus reducing citrate infusion, 2) Actively managing the patient’s circulation to correct hypoxia and shock. If the above treatments are ineffective, other anticoagulant methods should be considered.

***Insufficient base delivery***

Regardless of using 4% TSC solution or ACD-A as anticoagulant, CCC has the function of alkalizing plasma when it is completely metabolized in the body, that is, it is equivalent to a net input of alkali. These alkaline loads on the one hand buffer metabolic acidosis in renal injury patients and on the other hand supplement bicarbonate lost through the filter. If the net alkali load cannot meet the total demand of these two aspects, metabolic acidosis, due to insufficient citrate delivery, occurs. This type of metabolic acidosis is due to the lack of net base with normal anion gap (if there is no other metabolic acidosis causes present), which has a completely different mechanism during citrate accumulation. The following treatments are recommended: 1) Additional base supplements (e.g., 5% NaHCO_3_); 2) Reduce the flow rate of replacement fluid or dialysate to reduce the loss of CCC in the effluent (note the potential for an insufficient therapeutic dose); 3) Increase flow rate of citrate infusion (possibly simultaneously increase the blood flow rate).

***Therapeutic dose and metabolic complications***

As mentioned above, the main mechanism of metabolic acidosis or alkali poisoning complications of RCA is related to the amount of CCC entering the patient’s body and whether it can be adequately metabolized. Metabolic alkalosis can occur if excessive CCC enters the body and is completely metabolized, while metabolic acidosis occurs when the amount of CCC entering the body cannot be fully metabolized or insufficient base delivery partially caused by decreased CCC entering the body. The factors affected remove of CCC have influence on the acid–base balance. Such as semi-permeable membrane and therapeutic dose. The more CCC that is removed by the filter’s semi-permeable membrane, the less CCC will enter the body. At a certain blood flow rate, the clearance ratio of CCC through the semi-permeable membrane mainly depends on the replacement fluid or dialysate flow rate. The higher the flow rate of the replacement fluid or dialysate, the higher the CCC clearance ratio, and the less CCC remains in the patient’s serum.

When RCA is used, alkali required vary in different therapeutic doses. To maintain acid–base balance, it is necessary to calculate the additional amount of base supplementation based on the therapeutic dose and citrate infusion rate, and adjust it dynamically based on the results of blood gas analysis. Take an example for explanation. The available commercial hemofiltration basic replacement fluid (carbonate replacement fluid A, 4000 ml) is commonly used in China combined with 5% NaHCO_3_ (carbonate replacement fluid B, 250 ml) in order to compensate for the loss of base (See Additional file 1: Table S1 for more details), which means that when the replacement fluid is at a flow rate of 1000 ml/h, a supplement of 5% NaHCO_3_ at 63 ml/h is needed to maintain acid–base balance. One molecule of Citrate^3−^ entering the body will be metabolized into three molecules of HCO_3_^−^ when RCA is used. Taking 4% TSC as an example, if the flow rate is 200 ml/h, it is equivalent to 137 ml/h of net alkali (5% NaHCO_3_) supplementation before passing through filter. As shown in Table 8, at low replacement fluid flow rates, the demand of alkali supplementation is also at a lower level. Consequently, the net alkali load generated by RCA will result in metabolic alkalosis even without additional alkali supplementation. The alkali supplementation requirement increases with an increasing replacement fluid flow rate, consequently changing the net alkali load from positive to negative. Metabolic acidosis (normal anion gap) would occur if additional base is insufficiently supplemented (e.g., 5% NaHCO_3_). Therefore, when the citrate flow rate matches the blood flow rate, acid–base balance can be maintained with approximately 10 times replacement fluid flow rate to the citrate (4% TSC) flow rate. Metabolic alkalosis will occur when the replacement fluid or dialysate flow rate is significantly lower than this range, which can be corrected by increasing the replacement fluid or dialysate flow rate. Or on the contrary there will be metabolic acidosis, which requires additional alkaline supplementation (e.g., 5% NaHCO_3_) to maintain acid–base balance.

**Table 8:** The influence of the flow variation of carbonate replacement fluid on net alkali load at a specific 4% TSC flow rate

Replacement fluid flow rate corresponding to the requirement for alkali replenishment		Alkali supplements provided by RCA		Difference between alkali provided by RCA and alkali required^*^ (ml/h)
Replacement liquid A (ml/h)	Replacement liquid B (non-citrate anticoagulation) (ml/h)	4% TSC (ml/h)	Equivalent to supplementing alkali (ml/h)	
0	0	200	137	+ 137
1000	63	200	137	+ 74
1500	94	200	137	+ 43
2000	125	200	137	+ 12
2500	156	200	137	− 19
3000	188	200	137	− 51

This table is a mathematical simulation at a 4% TSC flow rate of 200 ml/h, in which the “alkali” flow rate is converted to 5% NaHCO_3_ (ml/h); Net alkali load = alkali replenishment demand subtracted by alkali produced by citrate metabolism (ml/h). For more information on the composition and concentration of replacement fluids A and B, please refer to Additional file 1. *RCA* regional citrate anticoagulation; *TSC* trisodium citrate; ^*^alkali excess when + ; alkali needed when -

##### Electrolyte disturbances

###### Recommendation 12

*Serum electrolyte levels, especially iCa and magnesium levels, need to be closely monitored for timely detection and treatment of electrolyte abnormalities. (A*greement degree: 97.6%, *Evidence quality III, Recommendation strength B).*

**Remarks**

***Hypocalcemia or hypomagnesemia***

Citrate in the extracorporeal circulation chelates iCa to form CCC, part of which along with some of the unchelated iCa will be lost in effluent through the semi-permeable membrane. Similarly, ionized magnesium can also be lost. If the missing ions are not properly replenished, hypocalcemia or hypomagnesemia will occur. Hypocalcemia reduces myocardial contractility, induces arrhythmia, tetany and other systemic symptoms. However, these complications are relatively easy to detect and manage with standard monitoring. It is recommended to monitor the iCa concentration in the filter and the blood in the body at least every 6 h. Total-Ca and total-Ca/iCa measurements are recommended once a day for patients at low risk of citrate accumulation and every 6 h for patients at high risk. The requirements for magnesium supplementation depend on the composition of the replacement fluid or dialysate being used. Thus, it is recommended to measure serum magnesium once a day.

***Hypercalcemia***

By following standard RCA therapy protocols and monitoring blood calcium levels, hypercalcemia is less likely to occur. In a few cases, such as citrate accumulation, increasing the amount of calcium supplement because of decreased in vivo iCa, is likely to cause high total-Ca and low iCa levels. This condition should be managed as previously discussed citrate accumulation.

Moreover, a retrospective study showed that in patients with severe hypercalcemia who underwent CVVH, RCA more effectively decreased calcium levels and had a superior filter lifespan and no obvious adverse events compared with low molecular weight heparin [58].

***Hypernatremia***

The impact of the altered sodium ion concentration caused by CRRT treatment needs to be considered in advance, especially when using RCA for anticoagulation. It should be noted that the increase of sodium ion may be more obvious in patients with hyponatremia, or the higher sodium might be beneficial to control brain edema.

The sodium concentration in 4% TSC solution or ACD-A is much higher than in normal serum (Table 2). There is a risk of hypernatremia from prolonged or high-dose citrate anticoagulants [25, 59]. However, hypernatremia is uncommon when following a standardized RCA protocol. The reasons for this include: 1) Although the sodium concentration in 4% TSC is high (the molar concentration is 408 mmol/L), the increased range of blood sodium concentration is not significant in patients with normal serum sodium within the commonly used parameters. 2) A considerable portion of sodium enters the effluent through the semi-permeable membrane when the sodium concentration is low in commonly used commercial replacement fluid (for example, the sodium concentration of carbonate base replacement fluid A is 113 mmol/L). If hypernatremia happens during RCA, blood sodium concentration can be reduced by increasing the replacement fluid or dialysate flow rate to allow more sodium clearance through the semi-permeable membrane. There is generally no need to reduce the citrate flow rate or adjust sodium concentration in the replacement/dialysate fluid.

However, the above discussion of uncommon hypernatremia in using RCA is based on the relatively low sodium concentration in replacement fluid A commonly used in China, whereas there are many commercial replacement/dialysate fluids outside China with a sodium content much higher than that (e.g., 140 mmol/L). When fluids with higher sodium concentrations is used, isosmolar citrate solutions could be used instead of ACD-A or 4% TSC in case occurrence of hypernatremia.

In brief, hypernatremia is not a contraindication for using RCA, however the effect on patients caused by the change of sodium concentration should be considered before apply.

#### Special issues during RCA

##### RCA and liver dysfunction

###### Recommendation 13

*Liver dysfunction is not recommended as a contraindication in using RCA. Patients with liver dysfunction are at increased risk for citrate accumulation and should be monitored closely during treatment. (A*greement degree: 92.9%, *Evidence quality II, Recommendation strength C).*

**Remarks**

It is generally believed that patients with liver dysfunction have an impaired ability to metabolize citrate [50]. Therefore, liver dysfunction has traditionally been considered a contraindication in using RCA [47]. However, several recent studies have shown that RCA can still be used safely in patients with liver dysfunction or even liver failure, without a significant increased risk of complications [57, 60]. In a prospective multicenter observational study (7 ICUs, 133 RCA-CRRT patients), patients were classified into three groups according to the levels of serum total bilirubin: ≤ 34.2 μmol/L for normal liver function, > 34.2 to ≤ 119.7 μmol/L for mild liver failure, and > 119.7 μmol/L for severe liver failure. The results showed no difference in the incidence of safety endpoints (severe acid–base disorders of any cause or iCa abnormalities) among the three groups, and RCA was well tolerated even in patients with severe liver failure [57]. A meta-analysis that included 10 observational studies of 1241 patients with hepatic dysfunction undergoing RCA-CRRT showed no significant difference in plasma citrate levels in patients compared to those without hepatic dysfunction [60]. Several studies have shown that RCA can also be used safely in patients with renal failure after liver transplantation or in patients being treated with extracorporeal artificial liver support [61–63].

There are two possible explanations for the above results. First, the liver is not the only place in the body that can metabolize citrate, but kidneys, muscles and other tissues that contain the needed enzymes also can metabolize citrate. Second, traditional liver function evaluation indexes [aspartate transaminase (AST), alanine transaminase (ALT), bilirubin, cholinesterase, Child–Pugh score, model of end-stage liver disease (MELD) score, etc.] have a poor ability to predict citrate accumulation [64]. Therefore, liver failure should not be regarded as a contraindication for RCA. These patients should be regarded as a special group, and the changes of iCa and other electrolytes should be closely monitored during treatment to identify those with citrate accumulation [25, 57, 60, 64–66]. It is possible to use a lower citrate flow rate combined with a lower blood flow while closely monitoring the ratio of serum total calcium to serum ionized calcium [25].

##### RCA and lactic acidosis

###### Recommendation 14

*Lactic acidosis is not recommended as a contraindication to RCA. But such patients are at increased risk of citrate accumulation and should be monitored closely during treatment. (A*greement degree: 92.9%, *Evidence quality II, Recommendation strength C).*

**Remarks**

CCC that enters the body is metabolized in the mitochondria through the tricarboxylic acid cycle, which is oxygen dependent. In patients with severe hypoxia, shock or mitochondrial dysfunction, citrate tends to accumulate because of the impaired tricarboxylic acid cycle induced by decreased mitochondrial oxidative chain activity. In theory, the use of RCA in these patients could be dangerous. However, clinical practice has shown that RCA use is well-tolerated in patients with shock, especially in those whose circulation was improving [53]. Therefore, there is still a lack of criteria for evaluating the severity of metabolic impairment and RCA contraindication in these patients. Lactic acid is an important marker of severe tissue hypoperfusion, severe hypoxia or mitochondrial dysfunction. Because lactate and citrate share the same metabolic pathways in mitochondria, citrate accumulation is often combined with hyperlactatemia. They are not causally related in pathophysiology, but important markers of severe metabolic impairment and disease severity. Such patients usually have a very high mortality rate [53, 64].

Retrospective studies showed that almost 100% of patients who develop citrate accumulation have lactic acidosis [52]. Lactate might be used to evaluate citrate metabolic capacity. However, in a significant proportion of patients with elevated lactate not associated with hypoxia or hypoperfusion, the tricarboxylic acid cycle in these patients may be expected to be normal without affecting citrate metabolism. Therefore, hyperlactatemia cannot be used as a diagnostic criterion for citrate accumulation, nor a contraindication for RCA. Even if there is a severe lactate accumulation due to hypoperfusion or hypoxia, RCA can be safely administered if lactic acid declines in the first few hours of treatment [53]. The study has shown that when a specific lactate threshold (e.g., 2.39 mmol/L) was used to assess the risk of citrate accumulation in RCA-CRRT patients, the negative predictive value was significant, and the positive predictive value was low [53]. Therefore, patients with persistent hyperlactatemia due to shock or hypoxia may have a higher risk of citrate accumulation than other patients, and such patients should be considered as a special group needing closer monitoring by measuring the total-Ca/iCa ratio and pH [53]. If total-Ca/iCa ratio exceeds 2.5, an intervention to reduce the citrate load or to discontinue the infusion should be considered [26].

##### RCA and CRRT modes

###### Recommendation 15

*RCA is not recommended to be used in slow continuous ultrafiltration (SCUF) mode, as CCC cannot be effectively removed. RCA can be safely used in other modes such as CVVH, CVVHD, and continuous veno-venous hemodiafiltration (CVVHDF). (A*greement degree: 95.2%, *Evidence quality III, Recommendation strength C).*

**Remarks**

The modes commonly used for CRRT include SCUF, CVVH, CVVHD, and CVVHDF. SCUF mode does not require replacement fluid, and the amount of effluent generated is much lower than in other modes. If RCA is used in the SCUF mode, CCC cannot be effectively removed by the semi-permeable membrane, and the excessive CCC entering the systemic circulation makes the patient prone to metabolic alkalosis [67]. In CVVH mode, RCA can be safely used regardless of pre- or post-dilution. The main disadvantage is that it is limited by transmembrane pressure and filtration fraction when it is necessary to increase the flow rate of replacement fluid to quickly remove CCC (such as in metabolic alkalosis or citrate accumulation). CVVHD mode eliminates solutes by diffusion without limitation of transmembrane pressure and filtration fraction, and can increase dialysate flow rate to increase the proportion of CCC clearance through the semi-permeable membrane to alleviate metabolic alkalosis or reduce citrate accumulation. RCA can also be safely used in CVVHD. The main limitation of CVVHD is that the citrate replacement fluid (balanced solutions) alone cannot be used, because the citrate replacement fluid should be infused pre filter in the extracorporeal circuit to exert anticoagulant effect and also as predilution replacement fluid, which should not be used as dialysate, as shown in Table 2. CVVHDF has the advantages of CVVH and CVVHD and can achieve higher clearance efficiency by increasing replacement fluid or dialysate flow rate, which is conducive to the management of metabolic complications of RCA. Table 9 shows RCA applications in different CRRT modes.

**Table 9:** Application of RCA in different CRRT modes

Mode	Solute removed	Replacement fluid or dialysate	Characteristics of CCC eliminated by semi permeable membrane	Feasibility of RCA
SCUF	Negligible	No	CCC cannot be effectively eliminated by semi-permeable membrane, and too much CCC entering the systemic circulation is prone to causing metabolic alkalosis	Not suitable
CVVH	Medium/small molecule	Yes	The flow rate of replacement fluid is limited by transmembrane pressure and filtration fraction	Yes
CVVHD	Small molecule	Yes	Not limited by transmembrane pressure and filtration fraction and can increase dialysate flow rate to increase CCC clearance through semi-permeable membrane. Citrate replacement solution cannot be used alone	Yes
CVVHDF	Medium/small molecule	Yes	Has advantages of both CVVH and CVVHD, and can achieve a larger replacement fluid or dialysate flow rate, which is conducive to the management of any metabolic complications of RCA	Yes

*RCA* regional citrate anticoagulation, *CRRT* continuous renal replacement therapy, *SCUF* slow continuous ultrafiltration, *CVVH* continuous veno-venous hemofiltration, *CVVHD* continuous veno-venous hemodialysis, *CVVHDF* continuous veno-venous hemodiafiltration

##### RCA and energy management

###### Recommendation 16

*In the energy management of patients with RCA-CRRT, the extra calories provided by this anticoagulant approach should be taken into account. However, due to the CRRT mode, treatment dose, different replacement fluids and different anticoagulant composition, the extra calories provided by RCA are difficult to accurately estimate. (A*greement degree: 95.2%, *Evidence quality III, Recommendation strength C).*

**Remarks**

Citrate is not only an intermediate product in the tricarboxylic acid cycle but also can be directly oxidized and decomposed. Complete oxidative decomposition of 1 mmol of citrate in the body can produce 0.59 kcal of heat [68, 69]. In addition, the additional glucose provided during anticoagulation with ACD-A also generates heat (complete oxidative decomposition of 1 mmol of glucose produces 0.73 kcal of heat). As shown in Table 10, the amount of energy substrate entering the body varies with the use of different citrate anticoagulant solutions and different replacement fluid or dialysate flow rates. Therefore, RCA can have an impact on patients’ caloric management. Anticoagulation alone can generate 350–900 kcal extra per day, which should not be ignored but be included in the overall nutritional management of patients. However, due to the CRRT mode, treatment dose, different replacement fluids and different anticoagulant composition, the extra calories provided by RCA are difficult to accurately estimate. In addition, the effect on energy management is more complex when different formulations of replacement fluid are used, such as lactate replacement fluid (complete oxidative decomposition of 1 mmol lactic acid produces 0.33 kcal) or high glucose replacement fluid [20, 70].

**Table 10:** Additional calories provided by anticoagulation with different citrate solutions

Item	4% TSC solution		ACD-A solution	
	Postdilution CVVH	CVVHD	Postdilution CVVH	CVVHD
*CRRT settings*				
Blood flow rate (ml/min)	150	100	150	100
Target concentration of citrate (mmol/L)	4	4	4	4
Citrate infusion rate (mmol/h)	36	24	36	24
Replacement fluid/dialysate flow rate (ml/h)	2000	2000	2000	2000
Clearance ratio of CCC (%)	31.75	47.62	31.75	47.62
*Energy substrates delivered to patient*				
Calcium citrate complex (mmol/h)	24.57	12.57	24.57	15.46
Glucose (mmol/h)			30.23	23.20
24 h total calories of RCA (kcal/24 h)	347.91	177.99	877.54	448.85

This table is calculated with a HCT = 30%, plasma flow rate = blood flow rate × (1—HCT), CCC sieving coefficient is 1. The clearance ratio of CCC = dialysis or replacement fluid flow rate/plasma flow rate. *TSC* trisodium citrate, *ACD-A* citrate-glucose anticoagulant A, *CVVHD* continuous veno-venous hemodialysis, *CVVH* continuous veno-venous hemofiltration, *CRRT* continuous renal replacement therapy, *CCC* citrate-calcium complexes, *RCA* regional citrate anticoagulation, *HCT* hematocrit

## Summary

RCA has many advantages over SHA and is recommended as the preferred anticoagulant method for CRRT. However, if the parameters are not set properly or monitoring is not standardized, metabolic acid–base and electrolyte disturbances can still occur. Citrate accumulation is a rare and fatal complication of RCA, but the higher risk of death may be primarily due to severe comorbid diseases rather than citrate accumulation itself. There are no absolute contraindications to using RCA. Shock, severe liver dysfunction, or hyperlactatemia should not be considered as absolute contraindications to RCA. These patients should be considered as belonging to a higher-risk group that should be monitored closely during treatment. Some CRRT modes (e.g., SCUF) are not suitable for RCA. In addition, RCA delivers additional energy substrates, which should be incorporated into overall daily nutrition management. Of course, many issues in clinical practice are very individual, and this guideline does not cover all RCA-related issues. As experience accumulates and evidence is added, the recommendation statements will be updated accordingly.

## Supplementary Information

**Additional file 1: Table S1**. Composition and concentration of commercial bicarbonate replacement fluid in China

## Data Availability

Not applicable.

## Abbreviations

ACD-A: Acid-citrate-dextrose formula A
ALT: Alanine transaminase
AST: Aspartate transaminase
CCC: Citrate-calcium complexes
CMDA: Chinese medical doctor association
CRRT: Continuous renal replacement therapy
CVVH: Continuous veno-venous hemofiltration
CVVHD: Continuous veno-venous hemodialysis
CVVHDF: Continuous veno-venous hemodiafiltration
GRADE: Grading of recommendations assessment, development and evaluation
HCT: Hematocrit
HIT: Heparin induced thrombocytopenia
iCa: Ionized calcium
KDIGO: Kidney disease improving global outcomes
MELD: Model of end-stage liver disease
RCA: Regional citrate anticoagulation
SCUF: Slow continuous ultrafiltration
SHA: Systemic heparin anticoagulation
SID: Strong ion difference
TSC: Trisodium citrate
