# Supplementary material for: Management of regional citrate anticoagulation for continuous renal replacement therapy: guideline recommendations from Chinese emergency medical doctor consensus
Source: Mil Med Res. 2023 May 29;10:23. doi: 10.1186/s40779-023-00457-9 (PMC10226261; doi:10.1186/s40779-023-00457-9)
Supplement: Supplementary file 1 — Additional file 1: Table S1. Composition and concentration of commercial bicarbonate replacement fluid in China [file 40779_2023_457_MOESM1_ESM.pdf]

Table S1 Composition and concentration of commercial bicarbonate replacement fluid in China

Replacement fluid composition	Concentration	
	mmol/L	mg/ml
Fluid A (4000 ml/bag)		
Glucose	10.6	1.91
Cl^-	118	4.18
Mg^{2+}	0.797	0.0194
Ca^{2+}	1.6	0.0639
Na^+	113	2.6
Fluid B (5% sodium bicarbonate solution, 250 ml/bag)		
HCO_3^-	595	36.3
Na^+	595	13.7

The commonly used replacement fluid formulation in China is bicarbonate replacement fluid, which consists of two independent parts: replacement fluid A and replacement fluid B. Replacement fluid A contains electrolytes close to serum concentration, but without alkali, and is commercially available in 4000 ml/bag. Replacement fluid B is a 5% sodium bicarbonate solution, used to supplement the alkali in the commercial formulation of 250 ml/bag. When using non-citrate anticoagulant CRRT, replacement fluids A and B are used in proportion, i.e., every 4 L of replacement fluid A to a consumption of approximately 250 ml fluid B (5% sodium bicarbonate solution), which will maintain acid-base balance in the body. However, in RCA-CRRT, citrate is metabolized to bicarbonate upon entry into the body, which corresponds to an additional base to the body. In this case, the flow rate of replacement fluid B (5% sodium bicarbonate) needs to be reduced according to the actual base requirements and acid-base status. *CRRT* continuous renal replacement therapy, *RCA* regional citrate anticoagulation
